# Quercetin promotes gastrointestinal motility and mucin secretion in loperamide-induced constipation of SD rats through regulation of the mAChRs downstream signal

**DOI:** 10.1080/13880209.2018.1474932

**Published:** 2018-06-28

**Authors:** Ji Eun Kim, Mi Rim Lee, Jin Ju Park, Jun Young Choi, Bo Ram Song, Hong Joo Son, Young Whan Choi, Kyung Mi Kim, Jin Tae Hong, Dae Youn Hwang

**Affiliations:** a College of Natural Resources & Life Science/Life and Industry Convergence Research Institute, Pusan National University, Miryang, Korea;; b Life Science Research Institute, Novarex Co., Ltd, Chungju, Korea;; c College of Pharmacy, Chungbuk National University, Chungju, Korea

**Keywords:** Gastrointestinal motility, mucosa layer, mucin, membrane water channel, IP3, antagonist

## Abstract

**Context:** Quercetin (QCT) has been known as a potential therapeutic strategy for gastrointestinal diseases because it contributes to the stabilization of mast cells, the prevention of histamine release and modulation of CaCC chloride channel.

**Objective:** We investigated the laxative effect and action mechanism of QCT in Lop-induced constipation model.

**Materials and methods:** Constipation of SD rats was induced by subcutaneous injection of loperamide (Lop) (4 mg/kg weight) in 0.5% Tween 20 twice a day for three days. After 24 h, the constipation group was further treated with 1× PBS (Lop + Vehicle treated group), 10 mg/kg of QCT (Lop + LQCT treated group), 20 mg/kg of QCT (Lop + MQCT treated group) or 40 mg/kg QCT (Lop + HQCT treated group) at once. At 24 h after QCT treatment, the constipation phenotypes were measured and the transverse colon was collected from SD rats.

**Results:** The gastrointestinal motility, the number of stools and histological structures were significantly recovered in Lop + QCT treated group compared with the Lop + Vehicle treated group. Also, above activity of epithelial cells and smooth muscle cells were regulated by the mRNA expression of the muscarinic acetylcholine receptors M2 and M3 (mAChR M2 and M3) and some mediators of their downstream signalling pathway. Finally, laxative effects of QCT on mAChR signalling pathway were significantly inhibited by the treatment of mAChR antagonist in primary smooth muscle of rat intestine cells (pRISMCs).

**Conclusions:** This study provides the first strong evidence that QCT can be considered an important candidate for improving chronic constipation induced by Lop treatment in animal models.

## Introduction

Quercetin (QCT) is an abundant flavonoid found in many fruits, vegetables and grains (Michael et al. 1992). Structurally, they have an oxygen-containing ring between two benzene rings, due to which they exist in several different forms in human body, such as QCT glycoside, QCT sulphate, QCT glucuronide and methylated QCT (Miles et al. 2014). QCT also affects a number of physiological processes and diseases including blood pressure, immune and neuronal functions, cancer, aging, obesity and diabetes (Miles et al. 2014). In the gastrointestinal tracts, QCT is especially known to stabilize the mast cells and prevent the release of histamine and other chemicals from them (Pearce et al. 1984). Also, QCT effectively modulate the activity of CaCC chloride channel in ileum and colon of mice during *ex vivo* and *in vivo* studies (Yu et al. 2016). However, to date, there is scant evidence for a direct role of QCT on gastrointestinal diseases such as diarrhoea, vomiting, gastroenteritis, constipation, irritable bowel syndrome (IBS) and celiac disease.

Several reports on extracts and compounds containing flavonoids have provided evidences for the possibility that flavonoid contributes to the improvement of constipation. Cactus (*Opuntia humifusa*) water extract contains high levels of flavonoids, and reportedly improves the fecal pellet number and water content, as well as the histological parameters (Han et al. 2017). Also, gastrointestinal disorders such as constipation, indigestion, dysentery and gastroenteritis were significantly improved with *Citrullus colocynthis* (L.) Schrad (bitter apple fruit) (Hussain et al. 2014). The crude extract of *Phyllanthus emblica* (Pe.Cr) and leaf aqueous extract of *Mareya micrantha* (Benth.) Müll. Arg. (Euphorbiaceae) shows high laxative activity and reduced the loperamide induced constipation (Meite et al. 2010; Najeeb-ur-Rehman et al. 2012; Mehmood et al. 2013). Furthermore, the methanol and hexane extracts of *Senna macranthera* leaves induce a laxative effect comparative to the standard drug bisacodyl (Guarize et al. 2012). Although previous studies have provided some information regarding the possibility of a correlation between some intestinal bowel diseases and flavonoid, no studies have investigated the laxative effects of a specific flavonoid in the constipated animal model.

The present study was therefore undertaken in the loperamide (Lop)-induced constipation model, to verify the possibility that considering various flavonoids, QCT is a key component for inducing laxative effects. Our results provide the first scientific evidence that QCT successfully improves the functional regulation of smooth muscle and epithelial cells in the transverse colon of the constipated animal model through the regulation of mAChRs signalling pathway.

## Materials and methods

### Experimental design for animal study

The animal protocol for the laxative effects of QCT was reviewed and approved based on the ethical procedures for scientific care set by the Pusan National University-Institutional Animal Care and Use Committee (PNU-IACUC; Approval Number PNU-2017-1493). Adult SD rats purchased from Samtako BioKorea Inc. (Osan, Korea) were handled in the Pusan National University-Laboratory Animal Resources Center, which is accredited by the Korea Food and Drug Administration (KFDA) (Accredited Unit Number-000231) and The Association for Assessment and Accreditation of Laboratory Animal Care (AAALAC) International (Accredited Unit Number; 001525). Animals were provided with *ad libitum* access to a standard irradiated chow diet (Samtako BioKorea Inc., Osan, Korea) and water. During the experiment, rats were maintained in a specific pathogen-free (SPF) state under a strict light cycle (lights on at 08:00 h and off at 20:00 h) at 23 ± 2 °C and 50 ± 10% relative humidity.

**Figure 1.**
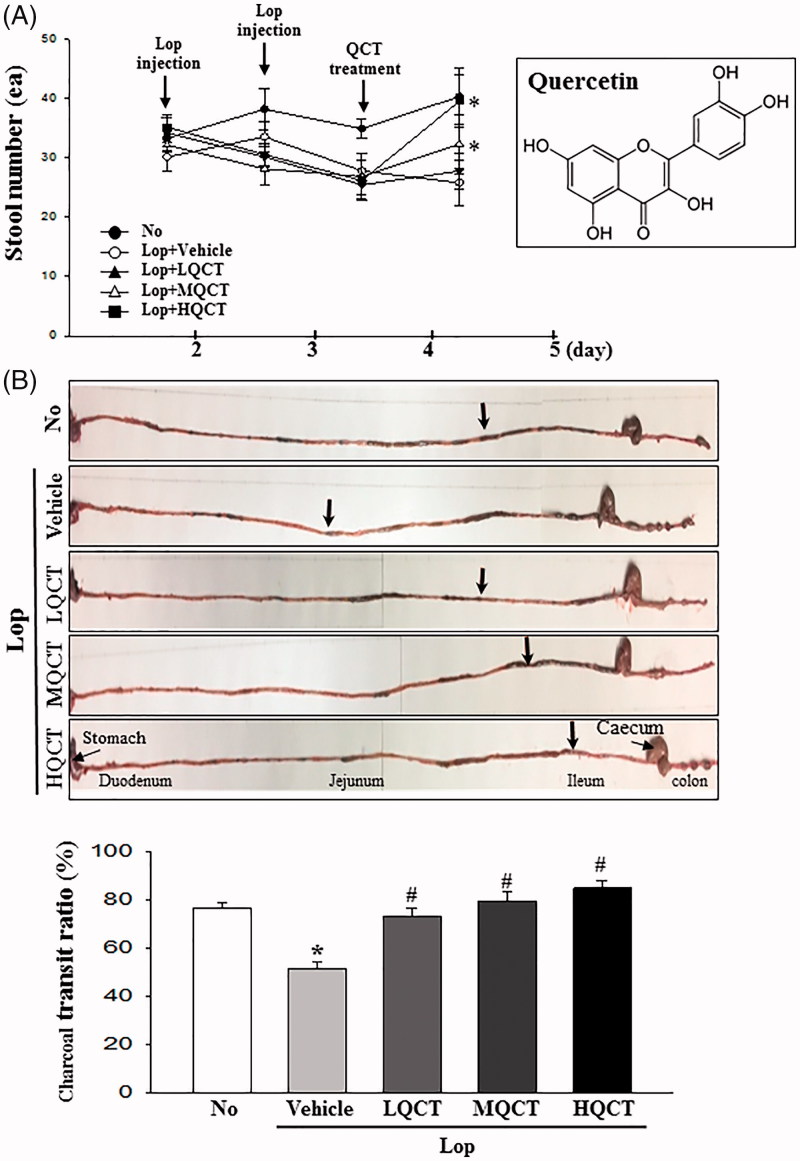
Alteration of stool number and gastrointestinal transit ratio. (A) Alteration of the stool number during the experimental period. The number of stools was measured in No, Lop + Vehicle and Lop + QCT treated groups. The experimental schedule consisted of two Lop injections and one QCT treatment for three days. Chemical structure of QCT is represented in right box. (B) Transit ratio of charcoal meal. Total intestinal tract was excised from mice of subset groups treated with charcoal meal powder. Their morphology was observed using a digital camera. Arrows indicate the potion of charcoal meal. The total distance travelled by the charcoal meal from the pylorus was measured. The charcoal meal transit ratio was then calculated, using the total length of intestine and distance of charcoal meal. Data represent the means ± SD of three replicates. *p < 0.05 compared to the No treated group. #*p* < 0.05 compared to the Lop + vehicle treated group.

Constipation of SD rats was induced by subcutaneous injection of Lop as the method described in Abbas et al. (2012) and Kim et al. (2016). First, 8-week-old SD rats (*n* = 35) were assigned to either a non-constipation group (No group, *n* = 7) or a constipation group (*n* = 28). Constipation was induced by subcutaneous injection of Lop (4 mg/kg weight) in 0.5% Tween 20 in saline twice a day for three days, whereas the non-constipation group was injected with 0.5% Tween 20 in saline alone. The No treated group was untreated during the experimental period at one time. Additionally, the constipation group was further divided into a Lop + Vehicle treated group (*n* = 7), a Lop + LQCT treated group (low dose QCT, *n* = 7), a Lop + MQCT treated group (meddle dose QCT, *n* = 7) and a Lop + HQCT treated group (high dose QCT, *n* = 7). Three Lop + QCT treated groups were orally administered 10, 20 or 40 mg/kg body weight QCT (Sigma-Aldrich Co., St. Louis, MO) (Figure 1(A)) at once, while the Lop + Vehicle treated group received the same volume of 1× PBS under the same pattern. At 24 h after QCT treatment, all SD rats were euthanized using CO_2_ gas, after which tissue samples were acquired and stored in Eppendorf tubes at –70 °C until assay.

### Analysis of food intake, water consumption and body weight

The food weight, water volume and body weight of SD rats treated with Vehicle or QCT were measured daily at 10:00 am throughout the experimental period using an electrical balance (for food and body weight) and a measuring cylinder (for water volume). The average food intake and water consumption were then calculated using the above data. All measurements were performed three times to ensure accuracy.

### Measurement of stool parameters and urine volume

During all experimental periods, SD rats of subset groups were bred in metabolic cages to harvest pure stools and urine to avoid any contamination (Daejong Instrument Industry Co., Ltd., Seoul, Korea). The stool number and weight were measured as previously described (Kim et al. 2013, 2016). Briefly, stools excreted from each SD rat were collected at 10:00 am, after which stool samples were weighed three times per sample using an electric balance and the number of stools was counted three times. The water content of stool was also analysed using the following formula:

Water content of stool = (A-B)/A × 100

where *A* is the weight of fresh stools collected from SD rats of subset groups and *B* is the weight of stools after drying at 60 °C for 12 h. Furthermore, urine volume was measured three times per sample using a cylinder.

### Gastrointestinal transit ratio

The gastrointestinal motility was measured by the method described by Choi et al. (2014) with some modifications. Animals were fasted for 18 h prior to the experiment, but were allowed to consume water *ad libitum*. Briefly, SD rats were fed with 1 mL of charcoal meal (3% suspension of activated charcoal in 0.5% aqueous methylcellulose) (Sigma-Aldrich Co., St. Louis, MO). After 30 min of treatment, these rats were euthanized using CO_2_ and the intestinal tract was collected from the abdominal cavity. The intestinal charcoal transit ratio was calculated as follows:

Charcoal transit ratio (%)=[(total small intestine length – transit distance of charcoal meal)/total small intestine length]×100

### Western blotting analysis

Total homogenate proteins were extracted from the transverse colons of all subset groups (No, Lop + Vehicle and Lop + QCT treated SD rats) using the Pro-Prep Protein Extraction Solution (Intron Biotechnology Inc., Seongnam, Korea). Following centrifugation at 13,000 rpm for 5 min, the protein concentrations were determined using a SMARTTM Bicinchoninic Acid Protein assay kit (Thermo Fisher Scientific Inc., Waltham, MA). Proteins (30 μg) were separated by 4–20% sodium dodecyl sulphate-polyacrylamide gel electrophoresis (SDS-PAGE) for 3 h, after which the resolved proteins were transferred to nitrocellulose membranes for 2 h at 40 V. Each membrane was then incubated separately with primary antibody, anti-Gα (Abcam, Cambridge, UK), anti-PI-3K (Cell Signaling Technology Inc., Cambridge, MA), anti-p-PI3K (Cell Signaling Technology Inc., Cambridge, MA), anti-PKC (Cell Signaling Technology Inc., Cambridge, MA), anti-p-PKC (Cell Signaling Technology Inc., Cambridge, MA) or anti-actin (Sigma-Aldrich Co., St. Louis, MO) overnight at 4 °C. The membranes were washed with washing buffer (137 mM NaCl, 2.7 mM KCl, 10 mM Na_2_HPO_4_, 2 mM KH_2_PO_4_ and 0.05% Tween 20), and incubated with horseradish peroxidase-conjugated goat anti-rabbit IgG (Zymed Laboratories, South San Francisco, CA) at a dilution of 1:1000 and room temperature for 2 h. Finally, the blots were developed using a Chemiluminescence Reagent Plus kit (Pfizer Inc., Gladstone, NJ). The signal image for each protein was acquired using the digital camera (1.92 MP resolution) of the FluorChem^®^ FC2 Imaging system (Alpha Innotech Corporation, San Leandro, CA) and their densities were semi-quantified using the AlphaView Program version 3.2.2 (Cell Biosciences Inc., Santa Clara, CA).

### Reverse transcription polymerase chain reaction (RT-PCR)

Total RNA was isolated from the frozen transverse colons using RNAzol B solution (Tet-Test Inc., Friendswood, TX) according to the manufacturer’s protocols. Following synthesis of cDNA, genes were amplified by subjecting the samples to 28 cycles of 30 s at 94 °C, 30 s at 62 °C and 45 s at 72 °C in a Perkin-Elmer Thermal Cycler. The primer sequences used to evaluate the level of mAChR M2 mRNA were as follows: sense primer, 5′-CCAGT ATCTC CAAGT CTGGT GCAAG G-3′, antisense primer, 5′-GTTCT TGTAA CACAT GAGGA GGTGC-3′. The primer sequences used to evaluate the level of mAChR M3 mRNA were as follows: sense primer, 5′-GTCAC TTCTG GTTCA CCACC AAGAG C-3′, antisense primer, 5′-GTGTT CACCA GGACC ATGAT GTTGT AGG-3′. The primer sequences used to evaluate the level of AQP8 mRNA were as follows: sense primer, 5′-GTAGT ATGGA CCTAC GTGAG ATCAA GG-3′, antisense primer, 5′-AGAAC CTTTC CTCTG GACTC ACCAC C-3′. The primer sequences used to evaluate the level of MUC2 mRNA were as follows: sense primer, 5′-GCTGC TCATT GAGAA GAACG ATGC-3′, antisense primer, 5′-CTCTC CAGGT ACACC ATGTT ACCAG G-3′. The sequences of the β-actin sense and antisense primers were 5′-TGGAA TCCTG TGGCA TCCAT GAAAC-3′ and 5′-TAAAA CGCAG CTCAG TAACA GTCCG-3′, respectively. The PCR products were quantified using 1% agarose gels and a Kodak Electrophoresis Documentation and Analysis System 120.

### Histopathological analysis

Transverse colons collected from No, Lop + Vehicle and Lop + QCT treated SD rats were fixed with 10% formalin for 48 h, embedded in paraffin wax, and then sectioned into 4 μm thick slices that were stained with haematoxylin and eosin (H&E, Sigma-Aldrich Co., St. Louis, MO). Morphological features of these sections were observed by light microscopy, after which the mucosa thickness, flat luminal surface thickness and number of goblet cells were measured using Leica Application Suite (Leica Microsystems, Heerbrugg, Switzerland).

For mucin staining, transverse colons collected from SD rats of subset groups were fixed with 10% formalin for 48 h, embedded in paraffin wax, and then sectioned into 4 μm thick slices that were subsequently deparaffinized with xylene and rehydrated. Next, the tissue sections on the slides were rinsed with distilled water and stained with an Alcian Blue Stain kit (IHC WORLD, Woodstock, MD). Finally, the morphological features in the stained colon sections were observed by light microscopy.

### Measurement of inositol phosphate (IP3) concentration

Levels of IP3 were determined using an IP3 ELISA kit (Cusabio Biotech Co., Ltd., Wuhan, China), according to the manufacturer’s instructions. The frozen transverse colon tissue was washed and homogenized in ice-cold 1X PBS (pH 7.2–7.4) using a glass homogenizer (Sigma-Aldrich Co., St. Louis, MO). The tissue lysates were centrifuged at 1000 rpm for 5 min at room temperature, after which the supernatant was collected for analysis. An anti-IP3 detection antibody was added and incubated at 37 °C for 60 min, after which the substrate solution was added and the samples were further incubated for 15 min at 37 °C. The reaction was terminated following the addition of stop solution, and the plates were read at an absorbance of 450 nm using a Molecular Devices VERSA max Plate reader (Molecular Devices, Sunnyvale, CA).

### Treatment of mAChR antagonist in pRISMC

Primary smooth muscle of rat intestine cells (pRISMCs) used in this study was prepared from intestines of infant rats (three days old) (*n* = 3) as previously described with some modification (Kim 2013). The purity of the pRISMCs population was confirmed by RT-PCR analysis of myosin-smooth muscle cells marker and neuronal cell marker, with some modification to a previously described method (Parajuli et al. 2010). To investigate the effects of mAChR antagonist, pRISMCs were seeded into 100 mm diameter culture dishes at a density of 1 × 10^7^ cells 10 mL^−1^, then incubated with 20 µM Lop (Sigma-Aldrich Co., St. Louis, MO) for 12 h at 37 °C. The culture media with Lop were subsequently discarded, and the cells were then incubated with 1× PBS (Vehicle), 10 µM ATR (Sigma-Aldrich Co., St. Louis, MO), 20 μM QCT or ATR + QCT for a further 12 h, as well as ATR + QCT for a further 6 h. These cells were then harvested by centrifugation at 3000 rpm for 10 min, and used for western blot analysis.

### Statistical analysis

One-way ANOVA (SPSS for Windows, Release 10.10, Standard Version, Chicago, IL) was used to determine the variance and identify significant differences between the No treated group and Lop treated groups, as well as between the Lop + Vehicle and Lop + QCT treatment within the constipation groups. All values are presented as the means ± standard deviation (SD). A *p* < 0.05 was considered significant.

## Results

### Effect of QCT treatment on feeding behaviour and excretion parameters

We first investigated whether QCT treatment improves the feeding behaviour and excretion of constipated SD rats. To achieve this, changes in the body weight, food intake, water consumption, stool parameter and urine volume were measured in Lop-induced constipated SD rats after administering three different doses of QCT. As shown in Table 1, no significant differences in the body weight and food intake were observed, although water consumption decreased in the Lop + MQCT and Lop + HQCT treated groups. However, the decrease in the number, weight and water content of stools was almost recovered in the Lop + MQCT and Lop + HQCT treated group (Figure 1(A) and Table 1). However, a reversal in the pattern was observed for urine volume. The increase in urine volume after Lop treatment significantly decreased after treatment with all three doses of QCT (Table 1). These results suggest that QCT treatment stimulates the excretion of stools in the Lop-induced constipation model without significant alteration of body weight and food intake.

**Table 1.** Alteration on the constipation parameters after QCT treatment.

		Lop			
Category	No	Vehicle	LQCT	MQCT	HQCT
Body weight (g)	309.1 ± 21.8	298.3 ± 11.1	303.0 ± 12.4	306.0 ± 20.7	306.6 ± 15.0
Food intake (g/day)	24.88 ± 2.9	24.50 ± 2.72	24.75 ± 2.88	24.19 ± 2.68	25.50 ± 3.49
Water consumption (mL)	19.50 ± 1.29	31.75 ± 1.71*	26.00 ± 2.31*	21.50 ± 1.91#	17.00 ± 2.16#
Stool number (ea)	40.25 ± 4.79	25.75 ± 3.86*	27.75 ± 2.99*	32.25 ± 4.99#	39.50 ± 4.43#
Stool weight (g)	04.60 ± 0.36	02.33 ± 0.28*	02.77 ± 0.48*	03.80 ± 0.70#	04.96 ± 0.78#
Stool water contents (%)	60.65 ± 2.93	34.78 ± 0.97*	36.50 ± 1.30*	49.90 ± 1.86*^,^#	72.20 ± 1.93#
Urine volume (mL)	09.75 ± 1.71	20.50 ± 2.65*	14.25 ± 1.71*^,^#	11.25 ± 3.30#	10.25 ± 2.87#

* *p* < 0.05 compared to the No treated group.

# *p* < 0.05 compared to the Lop + Vehicle treated-group.

### Effect of QCT treatment on gastrointestinal motility

To investigate whether the increase in the stools excretion was accompanied with changes in the gastrointestinal motility, the charcoal meal transit test was performed in Lop + QCT treated SD rats. As shown in Figure 1(B), the propulsion of charcoal meal decreased 33% in the Lop + Vehicle treated group compared with No treated group. After QCT treatment, the propulsion was completely recovered in a dose-dependent manner in all Lop + QCT treated groups. These results indicate that the increased excretion induced by QCT treatment is associated with increasing gastrointestinal motility in the Lop-induced constipation model.

### Effect of QCT treatment on the histology of transverse colon

**Figure 2.**
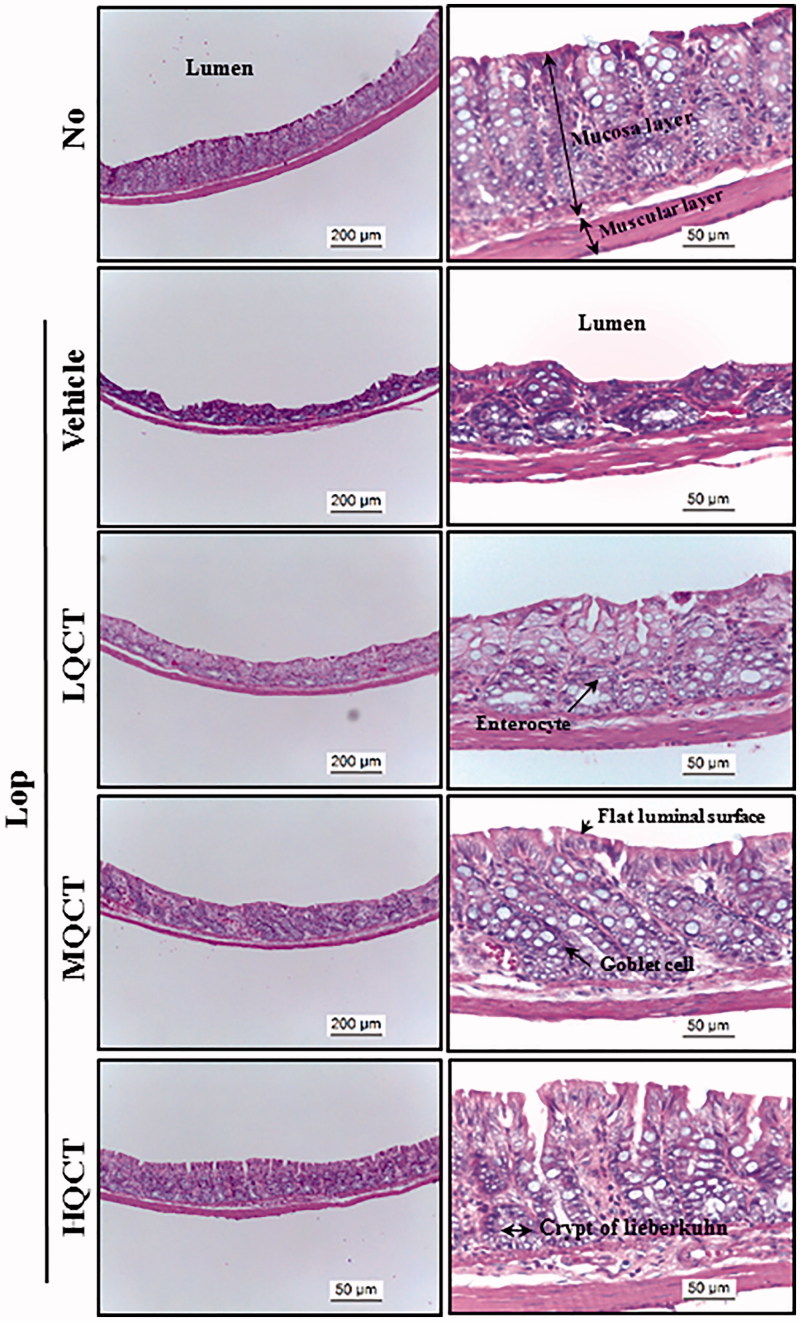
Histological structures of transverse colon in Lop-induced constipated rats after QCT treatment. H&E stained sections of transverse colon from the No, QCT, Lop + Vehicle or Lop + QCT treated groups were observed at 100 × (left column) and 400 × (right column) magnification using a light microscope. Five to six rats per group were assayed in triplicate by H&E. Histopathological parameters were determined using the Leica Application Suite (Leica Microsystems, Heerbrugg, Switzerland).

We further examined whether recovery of the excretion parameter due to QCT treatment reflects any histological alterations of the transverse colon induced by Lop treatment. To achieve this, alterations in the histological parameters that indicate laxative effects were evaluated in the transverse colons stained with H&E solution. The thickness of mucosa, muscle and flat luminal surface was significantly decreased in the Lop + Vehicle treated group relative to the No treated group. However, these alterations were dramatically recovered following the Lop + QCT treatment, when compared with the Lop + Vehicle treated group (Figure 2 and Table 2). Also, a significant decrease in the number of goblet cells and crypt of Lieberkuhn was observed in the Lop + Vehicle treated group compared with the No treated group. However, a dose-dependent recovery was observed in the Lop + QCT treated groups (Figure 2 and Table 2). These findings suggest that the abnormal structure of the transverse colon of Lop-induced constipated SD rats completely recovers due to QCT administration.

**Table 2.** Alteration on the histological parameter of constipated SD rats.

		Lop			
Categories	No	Vehicle	LQCT	MQCT	HQCT
Mucosa thickness (μm)	154.3 ± 14.8	72.6 ± 10.6*	111.3 ± 12.8*^,^#	133.2 ± 9.1#	147.3 ± 9.7#
Muscle thickness (μm)	51.2 ± 7.8	29.5 ± 8.4*	34.2 ± 9.1*^,^#	36.3 ± 8.4*^,^#	57.5 ± 8.2#
Flat luminal surface thickness (μm)	7.44 ± 0.7	4.15 ± 4.2*	5.41 ± 0.5*^,^#	7.08 ± 0.4#	9.5 ± 0.5#
Number of goblet cell (ea)	130.5 ± 8.4	58.4 ± 3.5*	82.1 ± 10.9*^,^#	125.1 ± 8.2#	142.3 ± 11.8#
Number of crypt of Lieberkuhn (ea)	17.2 ± 1.4	8.2 ± 1.1*	11.2 ± 0.9*^,^#	16.8 ± 2.0#	18.9 ± 1.9#

* *p* < 0.05 compared to the No-treated group.

# *p* < 0.05 compared to the Lop + Vehicle treated-group.

### Effect of QCT treatment on regulation of the ability to secrete mucin

**Figure 3.**
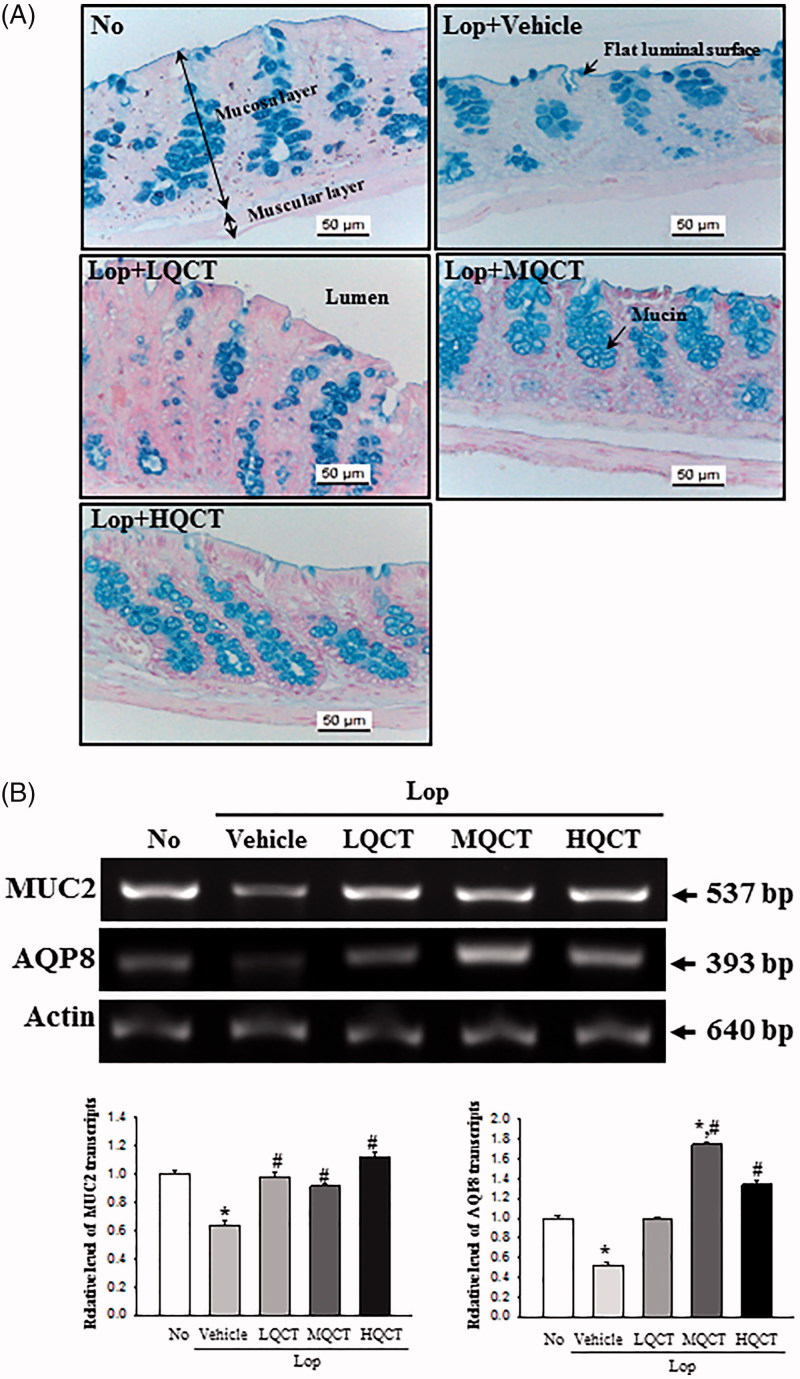
Detection of mucin secretion and membrane water channel expression in the transverse colon after QCT treatment. (A) Alteration of mucin level. Mucin secreted from crypt layer cells was stained with Alcian blue at pH 2.5, and images were observed at 400× magnification. Five to six rats per group were assayed in triplicate by Alcian blue staining. (B) The levels of MUC2 and AQP8 transcripts in the total mRNA of transverse colons were measured by RT-PCR using specific primers. After the intensity of each band was determined using an imaging densitometer, the relative levels of MUC2 and AQP8 mRNA were calculated based on the intensity of actin as an endogenous control. Five to six rats per group were assayed in triplicate by RT-PCR assays. Data represent the means ± SD of three replicates. *p < 0.05 compared to the No treated group. #*p*< 0.05 compared to the Lop + Vehicle treated group.

To investigate whether the laxative effects of QCT is accompanied by any changes in the ability to secrete mucin, the levels of mucin secretion and regulatory protein expression were measured in the transverse colon of SD rats. In the No treated group, mucin, as indicated by the dark blue stained region, was concentrated in the crypts in the mucosal layer of the transverse colon. The levels of mucin decreased in the Lop + Vehicle treated group compared with the No treated group. However, these levels were significantly increased in the Lop + MQCT and Lop + HQCT treated groups, while a constant was maintained in the Lop + LQCT treated group (Figure 3(A)). A similar pattern was observed in the expression levels of MUC2 mRNA, although a few differences were observed in the Lop + LQCT treated group (Figure 3(B)). At the same time, the level of AQP8 mRNA was measured to investigate whether mucin secretion induced by QCT treatment was accompanied by an altered expression of the membrane water channel. Similar expression levels of AQP8 mRNA and MUC2 were observed. Specifically, the levels increased 82%, 236% and 163% in the Lop + LQCT, Lop + MQCT and Lop + HQCT treated groups, respectively, compared to the Lop + Vehicle treated group (Figure 3(B)). These results indicate that the laxative effect of QCT may be associated with the ability to secrete mucin and expression of a membrane water channel in the transverse colon of Lop-induced constipated SD rats.

### Molecular mechanism of the gastrointestinal motility and mucin secretion ability after QCT treatment

**Figure 4.**
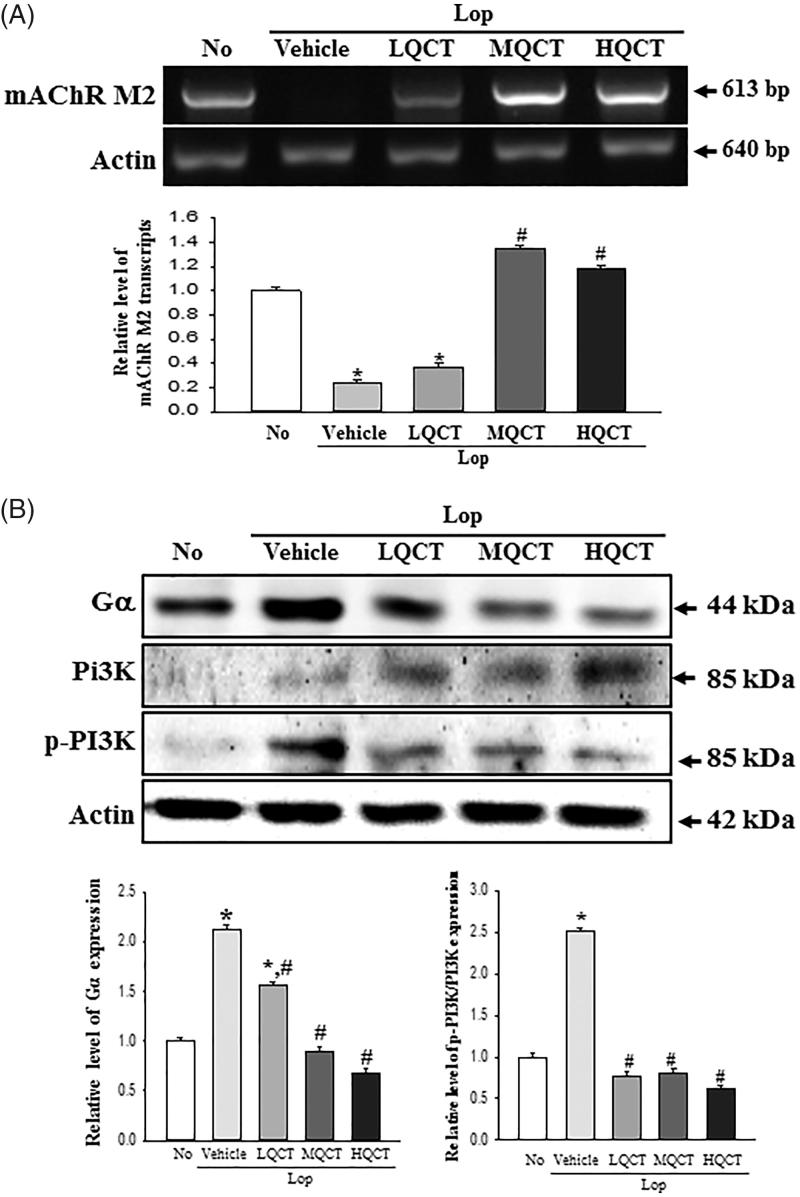
Expression of mAChR M2 and key mediators within their downstream signalling pathway in the transverse colon treated with QCT. (A) Expression level of mAChR M2. The levels of mAChR M2 transcripts in the total mRNA of transverse colons were measured by RT-PCR using specific primers. After the intensity of each band was determined using an imaging densitometer, the relative levels of mAChR M2 were calculated based on the intensity of actin. (B) Expression of key mediators within their mAChR M2 downstream signalling pathway. The expression of several related proteins, including Gα, PI3K and p-PI3K in the mAChR M2 signalling pathway, was measured by Western blot analysis using HRP-labelled anti-rabbit IgG antibody. After the intensity of each band was determined using an imaging densitometer, the relative levels of the four proteins were calculated based on the intensity of actin. Five to six rats per group were assayed in triplicate by Western blot and RT-PCR assays. Data represent the means ± SD of three replicates. *p < 0.05 compared to the No treated group. #*p* < 0.05 compared to the Lop + Vehicle treated group.

**Figure 5.**
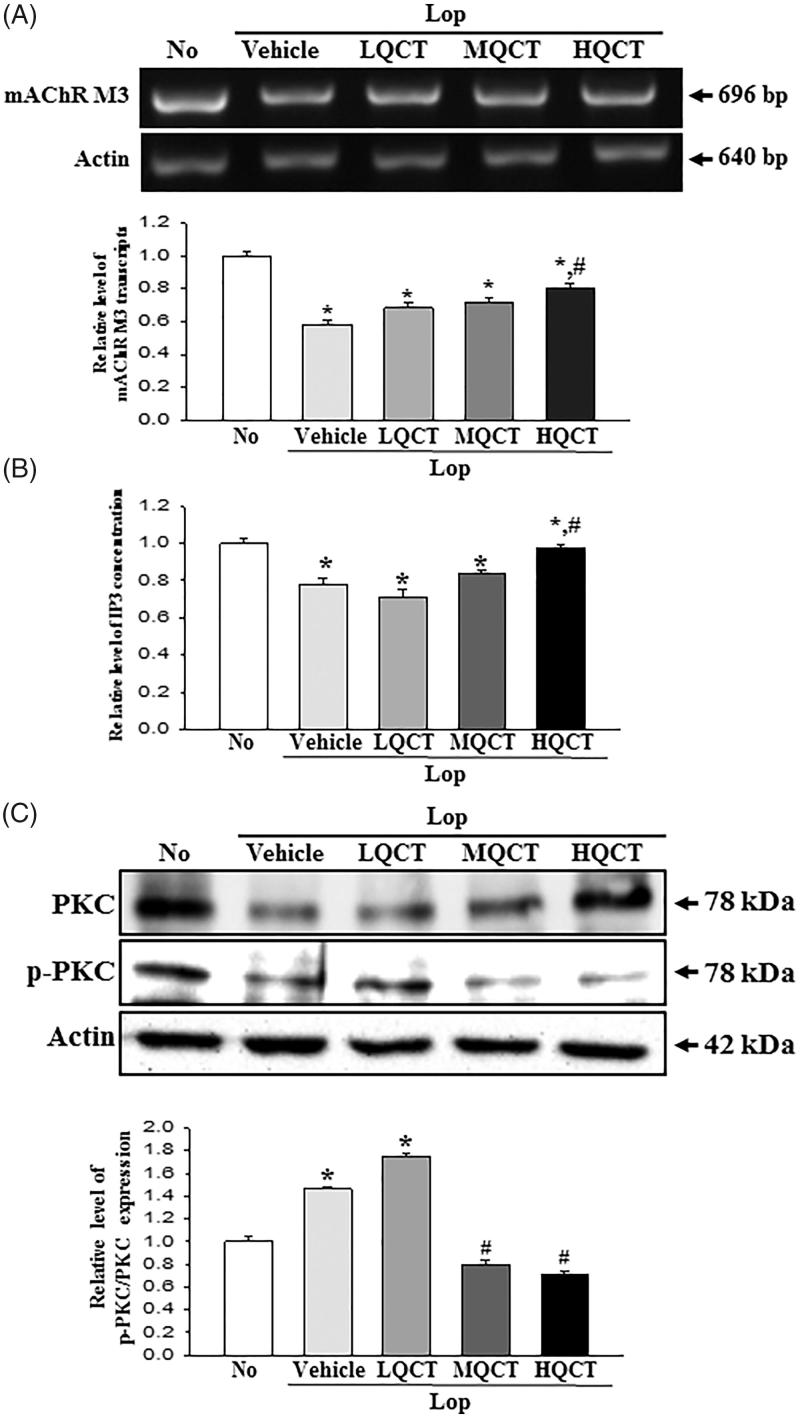
Expression of mAChR M3 and key mediators within their downstream signalling pathway in transverse colon treated with QCT. (A) Expression level of mAChR M3. The levels of mAChR M3 transcripts in the total mRNA of transverse colons were measured by RT-PCR using specific primers. After the intensity of each band was determined using an imaging densitometer, the relative levels of mAChR M2 were calculated based on the intensity of actin. (B) Concentration level of IP3. After the collection of the transverse colon for mice of each group, tissue lysates were prepared as described in section ‘Materials and methods’. The IP3 concentration in the total cell lysate was measured using an ELISA kit that detects IP3 at 5–1000 pg/mL. (C) Expression of PKC and p-PKC. The expression of several related proteins, including PKC and p-PKC in the mAChR M3 signalling pathway, was measured by Western blot analysis using HRP-labelled anti-rabbit IgG antibody. After the intensity of each band was determined using an imaging densitometer, the relative levels of the four proteins were calculated based on the intensity of actin. Five to six rats per group were assayed in triplicate by Western blot and RT-PCR assays. Data represent the means ± SD of three replicates. *p< 0.05 compared to the No treated group. #*p*< 0.05 compared to the Lop + Vehicle treated group.

Acetylcholine is a key regulator molecule for the functioning of smooth muscle and epithelial cells in the respiratory, gastrointestinal, cardiovascular and genitourinary organs (Kolahian and Gosens 2012; Kistemaker and Gosens 2015). To investigate the molecular mechanism for gastrointestinal motility and the ability to secrete mucin during the laxative action of QCT, changes in the expressions of mAChRs and key mediators within their downstream signalling pathway were measured in the transverse colon of the subset group. The subset group showed a similar pattern in the mRNA expression of mAChRs M2 and M3. The levels of these two receptors were lower in the Lop + Vehicle treated group than in the No treated group. However, a significant recovery in their expression was observed in the Lop + QCT treated group, although the ratio was greater in mAChRs M2 than M3 (Figures 4(A) and 5(A)). Furthermore, similar changes were observed in the expressions of key mediators of the mAChR downstream signalling pathway. In case of mAChRs M2 downstream signalling pathway, the levels of Gα expression and PI3K phosphorylation were higher in the Lop + Vehicle treated group. These levels dramatically recovered in the Lop + QCT treated group compared with the Lop + Vehicle treated group (Figure 4(B)). In the mAChRs M3 downstream signalling pathway, the IP3 concentration clearly reflected the expression level of mAChRs M3 (Figure 5(B)). However, the phosphorylation level of protein kinase C (PKC) showed a reverse trend of the mAChRs M3 expression and IP3 concentration. These levels were completely recovered in the Lop + MQCT and Lop + HQCT treated groups (Figure 5(C)). The results of this study suggest that the laxative effects of QCT are correlated with recovery of the down-regulation of mAChRs expression and their downstream signals in the transverse colons of constipated SD rats.

### Inhibition of QCT effects by mAChR antagonist treatment

**Figure 6.**
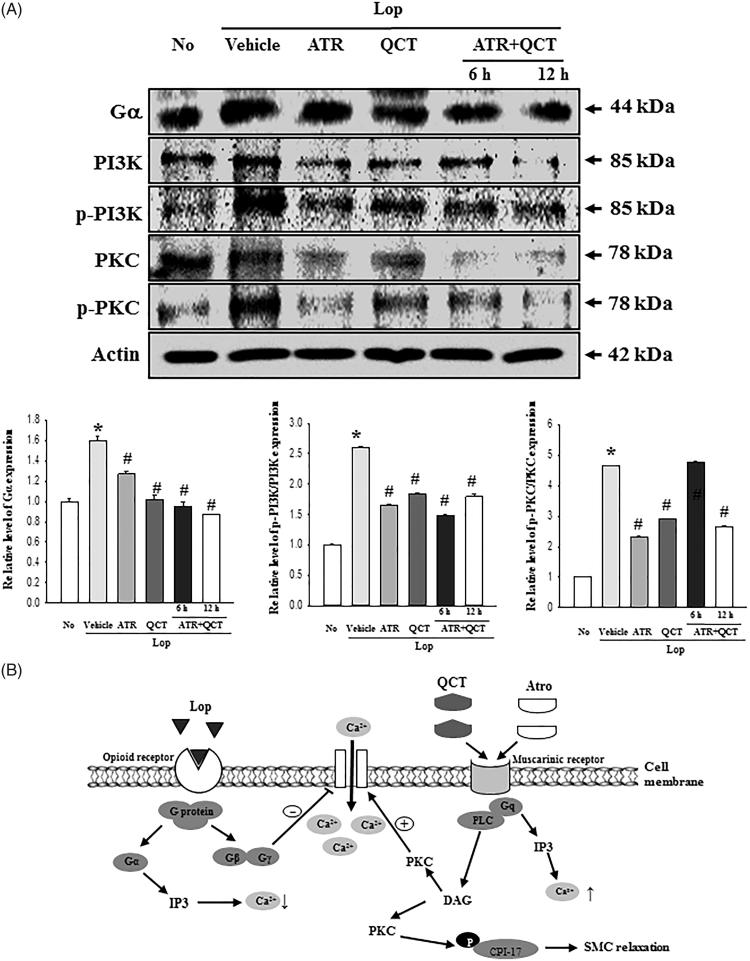
Inhibitory effects of mAChR M2 antagonist. (A) After treatment with Lop for 12 h, pRISMC were further incubated with QCT and ATR co-treatment, as described in section ‘Materials and methods’. The expression of several related proteins, including Gα, PI3K, p-PI3K, PKC and p-PKC in the mAChR signalling pathway, was measured by Western blot analysis using HRP-labelled anti-rabbit IgG antibody. Data represent the means ± SD of three replicates. *p< 0.05 compared to the No treated group. #*p*< 0.05 compared to the Lop + Vehicle treated group. (B) Suggested mechanism of QCT action in Lop-induced constipation. In this scheme, ATR effectively prevents the binding of QCT to muscarinic receptor.

Since QCT have a competitive binding affinity (*Ki* = 40–110 μM) to the human cloned mAChRs M1 (Swaminathan et al. 2014), we examined whether the laxative effects of QCT could be associated with the interaction between QCT and mAChR 2/3 in the transverse colon, since QCT recovery involved an alteration of the Lop-induced signal. To achieve this, the membrane expressions of the mAChRs signalling pathways were measured in pRISMC co-treated with ATR and QCT after Lop induction. The expression and phosphorylation of all three members showed a similar pattern in the subset group. Their level was higher in the Lop + Vehicle treated group than the No treated group. After the Lop + ATR or Lop + QCT treatment, these levels significantly decreased, although some differences were detected. Especially, the ATR pretreatment for 6 h and 12 h induced a further decrease in the Gα expression, PI3K phosphorylation and PKC phosphorylation in pRISMCs (Figure 6(A)). These results indicate that QCT regulates the gastrointestinal motility of smooth muscle cells and their ability to secrete mucin through binding the mAChRs.

## Discussion

Constipation is well known as an acute or chronic gastrointestinal disease characterized by infrequent bowel movements, hard and dry faeces, incomplete bowel evacuation and difficulty during defecation (Walia et al. 2009). Until now, several drugs were developed to treat this disease. In most cases, chemical drugs (laxatives) including Senna™, Correctol^®^, Exlax^®^, Senokot™ and Gaviscon^®^ act as stimulants to increase bulkiness and soften stool or as osmotic agents, trigger bowel movements and enhance water flow into the colon to promote elimination (Voderholzer et al. 1997). But, most of above drugs showed some undesirable side effects, such as myocardial infarction, artery contraction and coronary spasms (Lembo and Camilleri 2003; Busti et al. 2004; Kim et al. 2013). Therefore, many studies have focused on identifying novel laxatives with no side effects to treat constipation patients. As part of above studies, we investigated the laxative effect and action mechanism of QCT in Lop-induced constipation model. The results of the present study first demonstrated that QCT can improve the symptoms of constipation through the elevation of stool excretion, and the recovery of histological changes of the transverse colon in Lop-induced constipation model. Especially, our data show the laxative effects of QCT are tightly correlated with the interaction between QCT and mAChR M2/3 signalling pathway.

Stool excretion is considered an important factor during the developmental and mechanistic study of laxative drugs. The constipation model treated with Lop showed significant reduction of stool-related factors including number, weight and water content (Meite et al. 2010; Wintola et al. 2010; Kim et al. 2013, 2016; Lee et al. 2016). These alterations dramatically recovered with several plant extracts containing flavonoid. Previous studies have reported an increase in the number and water content of stools after the treatment with cactus (*Opuntia humifusa*) water extract and leaf aqueous extract of *Mareya micrantha* (Benth.) Müll. Arg. (Euphorbiaceae) having high flavonoid content (Meite et al. 2010; Han et al. 2017), although the treatment doses differed in each study. In the present study, a similar laxative effect on stool-related factors was observed in the Lop-induced constipation model treated with 20 or 40 mg/kg of QCT, although the highest effect was observed in the Lop + HQCT treated group. These results therefore provide novel evidence that various flavonoids can contribute to improving constipation, and are potential candidates for laxative drugs.

In the gastrointestinal tract, the smooth muscles generate motility of two kinds, after stimulation of action potential derived from interstitial cells of Cajal (Trowers and Tischler 2014). However, this motility is significantly delayed through the inhibition of repetitive non-propulsive contractions and high-amplitude propagated contractions following induction of constipation (Andrews and Storr 2011). Improved gastrointestinal motility was induced in the constipation model after treatment with natural products containing flavonoids. The intestinal transit ratio and the propulsion of charcoal meal were also significantly increased after treatment with Cactus (*Opuntia humifusa*), *Phyllanthus emblica* and *Senna macranthera* leaves (Meite et al. 2010; Guarize et al. 2012; Han et al. 2017). The alterations of gastrointestinal motility observed in the present study are very similar to those of previous studies; the QCT treatment induced the enhancement of charcoal transit ratio in a dose-dependent manner, as shown in Figure 2.

Histopatholgically, the transverse colons of animal models with constipation show several significant alterations. After Lop treatment, the average thickness of the transverse colon layer, the mucus layer, muscle and flat luminal surface is thinner than seen in the No treated groups (Kim et al. 2013, 2016). Also, the secretion of mucin declined in a similar pattern in the Lop + Vehicle treated group. However, these alterations completely recovered after treatment with some natural products (Meite et al. 2010; Kim et al. 2016). However, the laxative effects of natural products containing flavonoids were not focused on the alteration of the histological structure of the colon. Only the recovery of mucin secretion was induced by the treatment of Cactus (*Opuntia humifusa*) water extract with high flavonoid concentration (Han et al. 2017). In our study, the mucosa thickness, muscle thickness, flat luminal surface thickness, number of goblet cells and number of crypt of Lieberkuhn dramatically increased in the Lop + QCT treated group when compared with the Lop + Vehicle treated group. Also, the mucin secretion recovered after the QCT treatment. These findings are similar to those observed in animals treated with several flavonoid derived from natural extracts including Cactus (*Opuntia humifusa*) water extract, although the analysing factors were more diverse in our study.

Finally, the mAChRs mediate the actions of the neurotransmitter (ACh) in the central nerve system (CNS), peripheral nervous system (PNS) and the end organs of the parasympathetic nerves (van Koppena and Kaiser 2003; Ishii and Kurachi 2006). These receptors stimulate signal transduction pathways through the interactions with G protein, although five subtypes (M1–M5) were coupled with toxin-insensitive and toxin-sensitive G subtype protein (Caulfield 1993; Rümenapp et al. 2001). Furthermore, numerous compounds bind to the mAChRs as agonist including acetylcholine, nicotine, muscarine and epibatidine, as well as antagonist including atropine, pirenzepine and methoctramine (Ehlert et al. 2012). Two flavonoids, ombuin and QCT, possess high binding affinity to mAChR M1 (Ki = 40–110 µM) (Swaminathan et al. 2014). However, there are no reports on the interaction between QCT and mAChR during the improvement of constipation. In the present study, the mAChR expression and downstream signalling pathway dramatically recovered after the QCT treatment. Also, the stimulation of QCT was significantly prevented by the antagonist of mAChR in pRISMCs, as shown in Figure 6. These results therefore provide scientific evidence that the binding between QCT and mAChR plays a key role during the improvement of constipation induced with Lop injection. However, the current study regarding the inhibitory effects of mAChR antogonists (ATR) have some limitations and restrictions, for a clinical application of the results obtained from *in vitro* conditions to *in vivo* conditions.

Taken together, the results of this study suggest that QCT induces the recovery of stool excretion, gastrointestinal motility and histopathology in the Lop-induced constipation model. In addition, these results provide evidence that the laxative effects of QCT are mediated by the regulation of mAChRs and their downstream signals, as well as the secretion of mucin (Figure 6(B)). These findings further indicate that QCT could be considered a potential therapeutic candidate for the treatment of constipation, although many additional studies are required to confirm the above.
